# Quantitative Permeability MRI for Preoperative Differentiation of Warthin Tumors and Malignant Salivary Gland Lesions

**DOI:** 10.3390/diagnostics16152337

**Published:** 2026-07-25

**Authors:** Nicola Morelli, Elena Gianola, Marina Biondi, Claudia Caborni, Giuseppe Marchesi, Daria Salsi, Irene Herman, Domenico Cuda, Davide Colombi

**Affiliations:** 1 Neuroradiology Unit, Guglielmo da Saliceto Hospital, 29121 Piacenza, Italy; m.biondi@ausl.pc.it; 2 Department of Radiology, University of Parma, 43126 Parma, Italy; elena.gianola.b@gmail.com; 3 Radiology Unit, Guglielmo da Saliceto Hospital, 29121 Piacenza, Italy; c.caborni@ausl.pc.it (C.C.); g.marchesi@ausl.pc.it (G.M.); 4 Otolaryngology Unit, Guglielmo da Saliceto Hospital, 29121 Piacenza, Italy; d.salsi@ausl.pc.it (D.S.); i.herman@ausl.pc.it (I.H.); domenicorosario.cuda@unipr.it (D.C.); 5 Department of Diagnostic Imaging, Centro Diagnostico Rocca, 29121 Piacenza, Italy; colombidavide@gmail.com

**Keywords:** dynamic contrast-enhanced MRI, salivary gland tumors, pharmacokinetic modeling, Kep, Warthin tumor

## Abstract

**Background/Objectives**: Preoperative characterization of salivary gland tumors remains challenging because conventional MRI and diffusion-weighted imaging may show overlapping features, particularly between Warthin tumors and malignant lesions. This study evaluated the diagnostic value of quantitative permeability MRI based on dynamic contrast-enhanced pharmacokinetic analysis for differentiating salivary gland tumors. **Methods**: This retrospective single-center study included 51 patients with 59 histologically confirmed salivary gland lesions examined between January 2022 and November 2024. MRI included diffusion-weighted imaging and dynamic contrast-enhanced MRI (DCE-MRI). Quantitative pharmacokinetic analysis based on the Extended Tofts model provided Ktrans, Kep, Ve, and Vp. Interobserver reproducibility was assessed using the intraclass correlation coefficient, and diagnostic performance was evaluated by receiver operating characteristic analysis. **Results**: Of 59 lesions, 44 were benign, and 15 were malignant. Warthin tumors showed significantly higher Kep values than malignant lesions (median, 998 vs. 480 × 10^−3^/min; *p* = 0.002) and lower Ve values (median, 121 vs. 245 × 10^−3^; *p* = 0.004). ADC values partially overlapped between Warthin tumors and malignant lesions. Interobserver agreement was good, with ICC values ranging from 0.78 to 0.86. Kep showed the highest diagnostic performance in this cohort, with an AUC of 0.96. A Kep threshold of 723 × 10^−3^/min yielded 92.3% sensitivity and 93.3% specificity. **Conclusions**: Quantitative DCE-MRI, particularly Kep, may provide additional information for differentiating Warthin tumors from malignant salivary gland lesions within a multiparametric MRI assessment. The threshold identified in this cohort is exploratory and requires external validation before routine clinical use.

## 1. Introduction

Accurate preoperative characterization of focal lesions of the major salivary glands remains challenging in head and neck imaging [1,2,3]. Conventional magnetic resonance imaging (MRI) provides excellent soft tissue contrast and detailed morphologic assessment, but morphologic features alone are often insufficient to reliably distinguish benign from malignant salivary gland tumors because of substantial overlap in signal characteristics and enhancement patterns [2,4,5]. This limitation is particularly relevant for differentiating Warthin tumors from malignant lesions, which may show similar imaging appearances despite markedly different biologic behavior and therapeutic implications [6,7].

Diffusion-weighted imaging (DWI) is a well-established functional technique for salivary gland lesion assessment [8,9,10]. The apparent diffusion coefficient (ADC) reflects tissue cellularity, extracellular space, and stromal composition. Pleomorphic adenomas usually show high ADC values, whereas more cellular lesions tend to show lower diffusion values [8,9,11]. However, DWI has recognized limitations, especially in distinguishing Warthin tumors from malignant lesions, because both may show relatively restricted diffusion [9,10,12]. In addition, Warthin tumors and malignant lesions may both demonstrate rapid enhancement followed by wash-out, whereas pleomorphic adenomas more commonly show delayed progressive enhancement [13,14,15,16,17,18]. Therefore, the differentiation between Warthin tumors and malignant lesions represents a particularly relevant diagnostic challenge within multiparametric MRI assessment.

Dynamic contrast-enhanced MRI (DCE-MRI) provides complementary information on lesion vascularity and contrast kinetics [13,14,15]. Compared with semiquantitative time–intensity curve analysis, pharmacokinetic modeling allows a more objective assessment of tissue perfusion and permeability through parameters such as Ktrans, Kep, Ve, and Vp [14,15,16,17,18]. Among these, Kep has attracted particular interest because it may reflect extracellular contrast exchange and microvascular architecture, potentially improving differentiation between Warthin tumors and malignant lesions [16,17,18,19].

Accordingly, the primary aim of this study was to investigate whether quantitative DCE-MRI pharmacokinetic parameters could provide additional information for differentiating Warthin tumors from malignant salivary gland lesions, while considering DWI and semiquantitative DCE analysis as complementary components of the multiparametric assessment [6,13,18].

## 2. Materials and Methods

### 2.1. Patient Population

This retrospective single-center study was approved by the local Ethics Committee (AVEN, SIRER code 6747; approval date: 22 February 2025), which waived written informed consent because of the retrospective design. Patient selection was performed by systematic review of the institutional RIS and PACS databases. Between January 2022 and November 2024, 65 consecutive patients referred for MRI evaluation of focal salivary gland lesions were identified.

Exclusion criteria included lack of definitive pathologic confirmation, incomplete imaging studies, or insufficient image quality for quantitative analysis. Fourteen patients were excluded because definitive pathologic confirmation was unavailable. The final cohort consisted of 51 patients with 59 lesions. Eight patients had multiple synchronous lesions. Lesions were analyzed separately using a lesion-based approach. Histopathologic diagnosis served as the reference standard. Malignant histologic subtypes were pooled for the primary comparison because the study addressed the clinical distinction between Warthin tumors and malignant lesions and the number of cases within each subtype was insufficient for reliable separate analysis.

### 2.2. MRI Acquisition Protocol

MRI examinations were performed on a 1.5 T scanner (Ingenia Ambition; Philips Healthcare, Best, The Netherlands). The protocol included axial and coronal T2-weighted turbo spin-echo imaging with fat suppression, axial T1-weighted spin-echo imaging, and coronal short tau inversion recovery imaging. Diffusion-weighted imaging was acquired with single-shot echo-planar imaging using b-values of 0, 400, and 800 s/mm^2^, and ADC maps were generated automatically by monoexponential fitting.

Dynamic contrast-enhanced MRI was performed with a 3D T1-weighted spoiled gradient-echo sequence with fat suppression. Sequence parameters were as follows: TR, 4.0 ms; TE, 1.94 ms; flip angle, 15°; field of view, 250 mm × 250 mm; acquisition matrix, 140 × 180; acquisition voxel size, 1.4 mm × 1.8 mm × 3.5 mm; reconstructed voxel size, 1.0 mm × 1.0 mm × 3.5 mm; and 24 slices without interslice gap. Gadoteric acid (Dotarem, 0.5 mmol/mL) was administered intravenously at 0.1 mmol/kg with an injection rate of approximately 2 mL/s.

Precontrast T1 estimation used a dual flip-angle approach (8° and 12°), each sequence yielding one 3D volume in approximately 5 s. Dynamic imaging consisted of 60 sequential volumes with a temporal resolution of 4.9 s and a total scan time of approximately 5 min. After dynamic imaging, conventional post-contrast 3D T1-weighted fast field echo imaging with fat suppression was acquired for morphologic assessment of lesion enhancement.

### 2.3. Image Postprocessing and Semiquantitative Analysis

Postprocessing was performed with Philips IntelliSpace Portal software (Version 12.1; Philips Healthcare). A single manually drawn representative ROI was placed within the solid component of each lesion on the slice showing the largest cross-sectional tumor area, avoiding cystic, necrotic, and hemorrhagic regions. Semiquantitative analysis included time-to-peak, wash-out ratio, time–intensity curve classification, and area under the enhancement curve (AUC). AUC was reported in mM·s according to the native software output. Wash-out ratio was calculated as (SI_peak − SI_end)/SI_peak × 100.

Time–intensity curves were classified into three types according to time-to-peak and wash-out behavior: type A, delayed progressive enhancement with time-to-peak >120 s; type B, rapid early enhancement (time-to-peak ≤ 120 s) followed by marked wash-out (>30%); and type C, rapid enhancement (time-to-peak ≤ 120 s) followed by plateau or mild wash-out (<30%), according to previously described criteria [13,18].

### 2.4. Pharmacokinetic Analysis

Quantitative pharmacokinetic analysis was performed with the Extended Tofts model, a two-compartment pharmacokinetic model that describes contrast-agent exchange between the vascular plasma compartment and the extravascular extracellular space. This approach models the temporal behavior of gadolinium after intravenous injection by combining the arterial input function with tissue concentration–time curves derived from dynamic T1-weighted imaging. In this model, Ktrans represents the volume transfer constant from blood plasma to the extravascular extracellular space and is influenced by both tissue perfusion and microvascular permeability; Kep represents the reverse rate constant from the extravascular extracellular space back to plasma; Ve represents the fractional extravascular extracellular volume; and Vp represents the fractional plasma volume. Mathematically, Kep is related to Ktrans and Ve, reflecting the rate at which contrast agent returns from the extravascular extracellular space to the plasma compartment. These parameters provide complementary information on tumor vascularity, permeability, and extracellular space composition.

The arterial input function was determined automatically by the software using multiple arterial sampling points. Motion correction and curve smoothing were automatically applied according to the vendor implementation. Because quantitative pharmacokinetic modeling requires conversion of dynamic T1-weighted signal changes into gadolinium concentration–time curves, signal-to-concentration conversion was based on precontrast T1 estimation, a fixed hematocrit value of 0.42, and the contrast-agent longitudinal relaxivity coefficient. For gadoteric acid at 1.5 T, an r1 relaxivity value of 3.6 L·mmol^−1^·s^−1^ was used according to literature values. The resulting concentration–time curves were fitted according to the vendor implementation of the Extended Tofts model. Therefore, the absolute values of the derived parameters should be interpreted in relation to the acquisition protocol, contrast agent, field strength, and postprocessing implementation.

**Figure 1:**
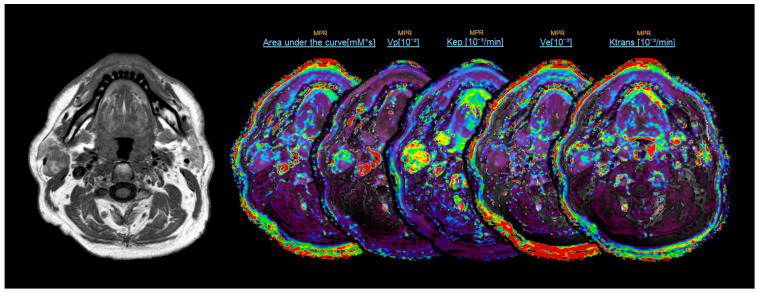
Extended Tofts Model–Derived Parametric Maps. Representative multiparametric quantitative DCE-MRI analysis of a right parotid lesion. Axial T1-weighted imaging is shown together with parametric maps derived from DCE-MRI, including area under the curve (AUC, mM·s), plasma volume fraction (Vp, ×10^−3^), reflux rate constant (Kep, ×10^−3^/min), extracellular extravascular volume fraction (Ve, ×10^−3^), and volume transfer constant (Ktrans, ×10^−3^/min). This panel illustrates the full set of quantitative permeability maps generated during the pharmacokinetic analysis of this Warthin tumor.

Values were reported according to the native vendor software output. Ktrans and Kep were expressed in ×10^−3^/min, whereas Ve and Vp were reported as unitless fractional parameters scaled in ×10^−3^. A representative example of Extended Tofts model-derived parametric maps is shown in Figure 1.

### 2.5. Interobserver Reproducibility Analysis

To assess interobserver reproducibility, a subset of 15 lesions was independently analyzed by a second radiologist with 10 years of experience in head and neck MRI who was blinded to clinical and histopathologic data. Primary measurements were performed by a neuroradiologist with 15 years of experience in head and neck imaging. ROIs were placed according to the same criteria used in the primary analysis.

Interobserver reproducibility for Ktrans, Kep, Ve, and Vp was assessed with the intraclass correlation coefficient (ICC) using a two-way random-effects model for absolute agreement.

Measurements from the primary reader were used for the final statistical analysis, whereas those from the second reader were used only for reproducibility assessment. ICC values < 0.50 indicated poor agreement, 0.50–0.75 moderate agreement, 0.75–0.90 good agreement, and >0.90 excellent agreement.

### 2.6. Statistical Analysis

Data distribution was assessed with the Shapiro–Wilk test. Because continuous variables were non-normally distributed, they were summarized as median (interquartile range). Lesions were analyzed individually using a lesion-based approach. Because eight patients contributed more than one synchronous lesion, an additional patient-level sensitivity analysis was performed by retaining only one lesion per patient, specifically the lesion with the largest axial diameter. The main comparison between Warthin tumors and malignant lesions and the ROC analyses were repeated in this reduced dataset. Group comparisons were performed with the Kruskal–Wallis test and Dunn correction for multiple comparisons. Diagnostic performance was evaluated with receiver operating characteristic analysis for quantitative imaging parameters showing significant differences between Warthin tumors and malignant lesions. Optimal cohort-specific thresholds were determined with the Youden index. For ROC analysis, Warthin tumors were defined as the positive class, with higher Kep values and lower Ve values considered indicative of Warthin tumors. Statistical significance was defined as *p* < 0.05. All analyses were performed with MedCalc software (Version 22.021; MedCalc Software Ltd., Ostend, Belgium).

## 3. Results

### 3.1. Study Cohort

The final cohort included 51 patients with 59 lesions. Eight patients presented with multiple synchronous lesions, whereas 43 had a single lesion. Median age was 64 years (interquartile range, 56–73 years), and there was a male predominance (34/51, 66.7%). Demographic characteristics are summarized in Table 1.

Histopathologic diagnoses included 44 benign lesions (74.6%) and 15 malignant lesions (25.4%). Warthin tumors were the most frequent benign lesions (26/59, 44.1%), followed by pleomorphic adenomas (15/59, 25.4%). The remaining benign lesions included 2 oncocytomas and 1 basal cell adenoma. Among malignant lesions, histologic subtypes included 5 mucoepidermoid carcinomas, 4 adenoid cystic carcinomas, 3 acinic cell carcinomas, 1 salivary duct carcinoma, and 2 adenocarcinomas not otherwise specified. Histopathologic distribution is detailed in Table 2.

### 3.2. Diffusion-Weighted Imaging

Diffusion-weighted imaging demonstrated characteristic differences among lesion types. Pleomorphic adenomas showed the highest ADC values, with a median of 1.59 (interquartile range, 1.44–1.77) × 10^−3^ mm^2^/s, significantly higher than all other groups (*p* < 0.001). Warthin tumors and malignant lesions showed partially overlapping ADC distributions, with median values of 0.88 (interquartile range, 0.78–0.99) × 10^−3^ mm^2^/s and 0.95 (interquartile range, 0.83–1.11) × 10^−3^ mm^2^/s, respectively. Additional benign lesions showed intermediate ADC values, with a median of 1.17 (range, 1.03–1.31) × 10^−3^ mm^2^/s.

### 3.3. Semiquantitative DCE Analysis

Semiquantitative DCE analysis demonstrated distinct enhancement patterns across lesion types. Pleomorphic adenomas predominantly exhibited type A curves (13/15, 86.7%), whereas Warthin tumors mainly showed type B kinetics (24/26, 92.3%). Malignant lesions displayed heterogeneous behavior, including type A (*n* = 2), type B (*n* = 6), and type C curves (*n* = 7). The distribution of time–intensity curve patterns is reported in Table 3.

### 3.4. Quantitative Permeability Analysis

**Figure 2:**
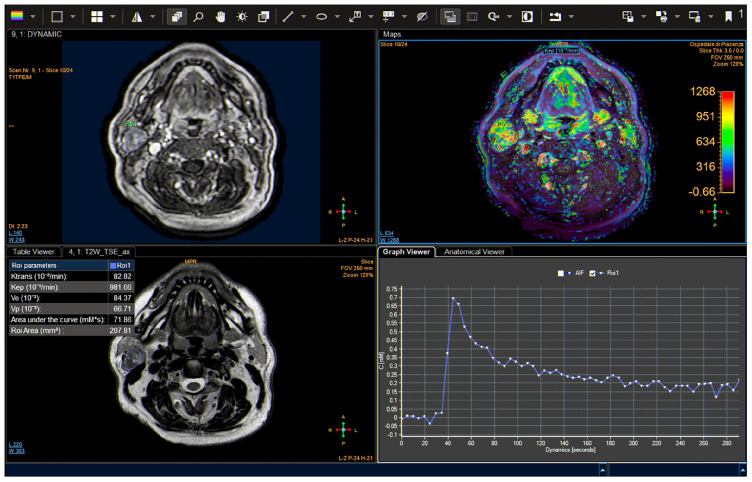
Representative multiparametric MRI findings in a Warthin tumor of the parotid gland. Multiparametric images, displayed using the clinical pharmacokinetic analysis interface, are organized as follows. (**Upper left**): dynamic contrast-enhanced acquisition. (**Lower left**): axial T2-weighted anatomical image demonstrating a solid lesion within the right parotid gland, with an overlaid table reporting quantitative pharmacokinetic parameters derived from ROI placement. (**Upper right**): color-coded Kep map overlaid on a contrast-enhanced T1-weighted image, showing the spatial distribution of Kep within the lesion and a measured value of 981 × 10^−3^/min. (**Lower right**): corresponding time–intensity curve showing rapid early enhancement followed by marked wash-out, consistent with a type B curve.

**Figure 3:**
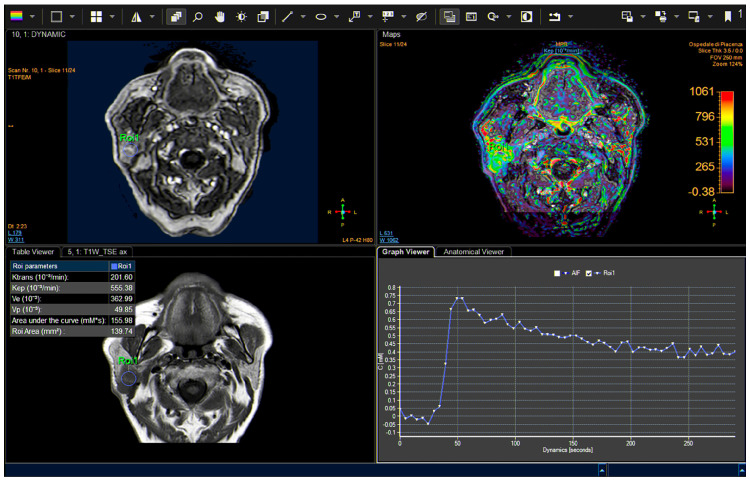
Representative multiparametric MRI findings in a malignant salivary gland lesion. (**Upper left**): dynamic contrast-enhanced acquisition. (**Lower left**): axial T1-weighted anatomical image demonstrating a solid lesion within the right parotid gland. (**Upper right**): color-coded Kep map overlaid on a contrast-enhanced T1-weighted image, showing the spatial distribution of Kep within the lesion and a measured value of 555 × 10^−3^/min. (**Lower right**): corresponding time–intensity curve showing rapid early enhancement followed by wash-out, consistent with a type B curve. Although this lesion and the Warthin tumor shown in Figure 2 both exhibited type B kinetics, their quantitative Kep values differed, illustrating the additional diagnostic information provided by pharmacokinetic analysis beyond curve classification.

Quantitative permeability assessment showed the most pronounced differences between Warthin tumors and malignant lesions. Representative examples of vendor-based DCE-MRI displays, including morphologic imaging, time–intensity curves, and Kep maps, are shown in Figure 2 and Figure 3. These figures illustrate the complete postprocessing workflow. Quantitative ROI measurements were interpreted together with morphologic MRI findings, diffusion imaging, and time–intensity curves rather than by visual inspection of the color maps alone.

**Figure 4:**
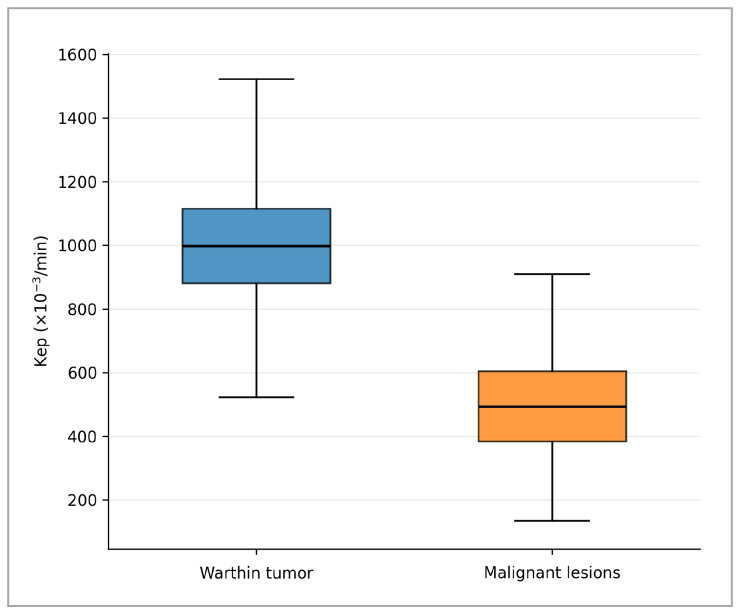
Boxplot representation of Kep values in Warthin tumors and malignant salivary gland lesions. Boxes represent interquartile ranges, horizontal lines indicate median values, and whiskers indicate the spread of observed values. Limited overlap between groups is observed. The difference in Kep values between groups was statistically significant (*p* = 0.002).

Warthin tumors showed markedly higher Kep values than malignant lesions, with median values of 998 (IQR, 870–1135) versus 480 (IQR, 390–605) × 10^−3^/min, respectively (*p* = 0.002). Ve values were significantly lower in Warthin tumors than in malignant lesions, with median values of 121 (IQR, 97–145) versus 245 (IQR, 203–300) × 10^−3^, respectively (*p* = 0.004). Figure 4 shows the distribution of Kep values, with limited overlap between groups.

Across lesion types, Ktrans and Vp showed substantial overlap, with no significant differences among groups (all *p* > 0.05). Median Ktrans and Vp values were 212 (IQR, 170–274) × 10^−3^/min and 75 (IQR, 59–92) × 10^−3^ in Warthin tumors, 188 (IQR, 145–238) × 10^−3^/min and 62 (IQR, 49–80) × 10^−3^ in malignant lesions, and 140 (IQR, 104–184) × 10^−3^/min and 50 (IQR, 37–65) × 10^−3^ in pleomorphic adenomas. Other benign lesions showed median Ktrans and Vp values of 172 × 10^−3^/min (range, 138–210) and 58 × 10^−3^ (range, 43–72), respectively. Quantitative and semiquantitative imaging parameters are summarized in Table 4.

### 3.5. Interobserver Reproducibility

Interobserver reproducibility for quantitative permeability parameters showed good overall agreement. ICC values were 0.81 (95% CI, 0.66–0.91) for Ktrans, 0.86 (95% CI, 0.73–0.93) for Kep, 0.78 (95% CI, 0.60–0.89) for Ve, and 0.80 (95% CI, 0.64–0.90) for Vp.

### 3.6. Diagnostic Performance

Receiver operating characteristic analysis identified Kep as the parameter with the highest diagnostic performance for differentiating Warthin tumors from malignant lesions, with an AUC of 0.96 (95% CI, 0.91–0.99). Ve also showed good discriminatory performance, with an AUC of 0.87 (95% CI, 0.78–0.94). The optimal threshold for Kep was 723 × 10^−3^/min, yielding 92.3% sensitivity and 93.3% specificity, whereas the optimal threshold for Ve was 180 × 10^−3^, yielding 80.8% sensitivity and 86.7% specificity. Diagnostic performance metrics are summarized in Table 5.

To assess the effect of multiple lesions in the same patient, a sensitivity analysis was performed using one lesion per patient. This analysis included 51 observations, with 19 Warthin tumors and 15 malignant lesions in the main comparison. Kep remained significantly higher in Warthin tumors than in malignant lesions, with median values of 1005 × 10^−3^/min (IQR, 880–1120) and 480 × 10^−3^/min (IQR, 390–605), respectively (*p* = 0.006). Ve remained significantly lower in Warthin tumors, with median values of 120 × 10^−3^ (IQR, 98–145) and 245 × 10^−3^ (IQR, 203–300), respectively (*p* = 0.021). Kep retained high diagnostic performance, with an AUC of 0.95 (95% CI, 0.87–0.99). A threshold of 720 × 10^−3^/min yielded 89.5% sensitivity and 93.3% specificity. Ve showed an AUC of 0.85 (95% CI, 0.72–0.94), with 78.9% sensitivity and 86.7% specificity at a threshold of 180 × 10^−3^.

## 4. Discussion

Quantitative permeability MRI provided additional information for salivary gland tumor characterization beyond diffusion imaging and semiquantitative perfusion analysis. Overall, our findings support the concept that diffusion- and permeability-derived metrics reflect different biologic properties of tumor tissue and should be considered complementary components of a multiparametric approach [6,18,20,21].

Diffusion-weighted imaging findings were in line with previous reports, with pleomorphic adenomas showing the highest ADC values and partial overlap between Warthin tumors and malignant lesions [8,9,10,12]. Similar observations were reported in the systematic review by Coudert et al., which highlighted the value of ADC for identifying pleomorphic adenomas while emphasizing its more limited performance in distinguishing Warthin tumors from malignant lesions [6].

Semiquantitative DCE analysis reproduced the expected enhancement patterns: pleomorphic adenomas showed delayed progressive enhancement, whereas Warthin tumors demonstrated rapid enhancement followed by marked wash-out. Malignant lesions displayed more heterogeneous kinetic behavior. This reinforces prior evidence from Yabuuchi et al. showing that time–intensity curve analysis is clinically informative but insufficient as a standalone tool, thereby supporting the added value of pharmacokinetic modeling [13,18].

The most relevant findings emerged from quantitative permeability analysis, particularly in the comparison between Warthin tumors and malignant lesions. Warthin tumors demonstrated markedly elevated Kep values together with significantly lower Ve values than malignant lesions. This permeability profile parallels previous observations by Yabuuchi et al., Huang et al., and Mungai et al., who reported higher Kep values in Warthin tumors than in other salivary gland neoplasms [18,19,22].

From a physiologic perspective, Kep should not be interpreted as a direct measure of microvascular permeability alone. In the Extended Tofts model, Kep represents the rate constant describing contrast return from the extravascular extracellular space to plasma and depends on both Ktrans and Ve. Therefore, the combination of higher Kep and lower Ve observed in Warthin tumors is consistent with rapid contrast exchange within a relatively small extracellular compartment, fitting well with their characteristic oncocytic epithelium, dense lymphoid stroma, and rich capillary network [19]. By contrast, malignant lesions showed lower Kep and higher Ve values, possibly reflecting a larger and more heterogeneous extracellular compartment together with more complex microvascular architecture [19,22]. The histologic heterogeneity of the malignant group may also have contributed to these findings.

The present study addressed a specific diagnostic challenge rather than the general classification of benign and malignant salivary gland tumors. Pleomorphic adenomas usually showed higher ADC values together with delayed progressive enhancement and therefore were less likely to overlap with malignant lesions. In contrast, Warthin tumors frequently showed restricted diffusion and rapid wash-out kinetics similar to those of malignant tumors, making this differential diagnosis clinically more demanding. Consequently, quantitative permeability parameters should be regarded as complementary to morphologic MRI, DWI, and semiquantitative DCE analysis and should not be interpreted as independent diagnostic criteria. MRI findings may support preoperative characterization and clinical decision-making, but histopathological examination remains the definitive reference standard for diagnosis. This conclusion applies only to primary salivary gland lesions, as lymph nodes were not included in the present analysis; therefore, no conclusions can be drawn regarding nodal characterization.

In our dataset, Kep showed the highest diagnostic performance for differentiating Warthin tumors from malignant lesions. This result is concordant with previous quantitative DCE-MRI studies in which Kep emerged as one of the most informative pharmacokinetic parameters for salivary gland lesion differentiation [18,19,21,22,23]. Although Ve also showed diagnostic capability, its performance remained lower than that of Kep, with an AUC of 0.87 compared with 0.96. Thus, Ve appears to contribute to lesion characterization, but with less discriminatory strength in this specific diagnostic setting.

However, the observed AUC should be interpreted cautiously because of the relatively limited sample size and the need for external validation. The optimal cohort-specific thresholds identified in this study, namely 723 × 10^−3^/min for Kep and 180 × 10^−3^ for Ve, should therefore be considered exploratory and potentially platform-specific rather than universally applicable. Previous investigations have shown variability in optimal threshold values across institutions and software implementations, likely reflecting differences in acquisition protocols, pharmacokinetic modeling, and postprocessing strategies [18,22,24].

Ktrans showed broader overlap among lesion groups, supporting the view that this parameter may be less specific than Kep for differentiating salivary gland tumors [21,22,23,25]. This lower discriminatory power may be related to the fact that Ktrans reflects the combined effects of vascular permeability and perfusion, particularly in lesions with heterogeneous vascular characteristics [18,25].

Taken together, these results support the added value of permeability-derived parameters in conjunction with diffusion imaging. Whereas ADC reflects tissue cellularity and extracellular structural characteristics, pharmacokinetic parameters provide additional information on microvascular permeability and contrast dynamics. Within this multiparametric framework, permeability MRI may be particularly useful when the differentiation between Warthin tumors and malignant lesions is clinically relevant [6,18,19].

From a methodological perspective, quantitative DCE-MRI parameters depend on several technical factors, including temporal resolution, arterial input function estimation, pharmacokinetic model implementation, and postprocessing strategy [26,27,28]. These factors may influence absolute quantitative values and contribute to variability across studies and imaging platforms [27,29]. As emphasized by recommendations from the Quantitative Imaging Biomarkers Alliance, standardization of acquisition protocols, temporal resolution, and pharmacokinetic modeling strategies is essential to improve reproducibility and facilitate cross-study comparisons [26]. In addition, multi-algorithm comparison studies have shown that differences in arterial input function modeling, temporal sampling, and fitting approaches may substantially affect calculated permeability parameters [28,29].

This study has several limitations. First, this was a retrospective single-center study, and although the number of malignant lesions reflected routine clinical practice, the overall sample size remained limited, particularly the number of malignant lesions (*n* = 15). Larger multicenter cohorts are therefore required to validate the present findings and the proposed thresholds. Second, malignant lesions comprised different histologic subtypes that were pooled because subgroup analysis was not feasible. Manual ROI placement may also have introduced variability despite good interobserver agreement, particularly in heterogeneous lesions. Although the primary analysis was lesion-based, the sensitivity analysis using one lesion per patient produced results consistent with the main findings. In addition, the ROC performance and thresholds were derived and evaluated in the same cohort and may therefore overestimate diagnostic performance. Finally, the vendor-specific pharmacokinetic implementation may limit direct comparison of absolute parameter values across different imaging platforms [30].

Overall, quantitative DCE-MRI, particularly Kep, may provide additional information when Warthin tumors and malignant lesions remain difficult to distinguish using morphologic MRI, DWI, and semiquantitative DCE analysis. Its potential role is adjunctive, and further validation in larger multicenter cohorts and across imaging platforms is required before routine clinical use.

## 5. Conclusions

Quantitative DCE-MRI, particularly Kep, may support the differentiation of Warthin tumors from malignant salivary gland lesions within a multiparametric MRI assessment. Its role remains adjunctive and should be integrated with clinical, morphologic, and pathological findings. The threshold identified in this cohort requires external validation before clinical use.

## Figures and Tables

**Table 1:** Study population and demographic characteristics of the final cohort.

Variable	Value
Patients initially identified	65
Excluded patients	14
Included patients	51
Total lesions analyzed	59
Patients with multiple synchronous lesions	8
Patients with a single lesion	43
Median age, years (IQR)	64 (56–73)
Male sex, *n* (%)	34 (66.7%)

Note. Values are reported as number of patients or lesions, percentage, or median with interquartile range (IQR), as appropriate.

**Table 2:** Histopathological distribution of the analyzed salivary gland lesions.

Lesion Category/Histological Subtype	*n*	% of Total Lesions
**Benign lesions (overall)**	44	74.6
— Warthin tumor	26	44.1
— Pleomorphic adenoma	15	25.4
— Other benign lesions	3	5.1
**Malignant lesions (overall)**	15	25.4
— Mucoepidermoid carcinoma	5	8.5
— Adenoid cystic carcinoma	4	6.8
— Acinic cell carcinoma	3	5.1
— Salivary duct carcinoma	1	1.7
— Adenocarcinoma NOS	2	3.4

Note. Overall benign and malignant lesions and individual histological subtypes are reported as absolute numbers and percentages of the total lesion cohort.

**Table 3:** Distribution of time–intensity curve (TIC) patterns according to lesion type.

Lesion Type	Type A	Type B	Type C
Pleomorphic adenoma (*n* = 15)	13 (86.7%)	1 (6.7%)	1 (6.7%)
Warthin tumor (*n* = 26)	0	24 (92.3%)	2 (7.7%)
Malignant lesions (*n* = 15)	2 (13.3%)	6 (40.0%)	7 (46.7%)

Note. Type A indicates delayed progressive enhancement, type B rapid enhancement with wash-out, and type C plateau or intermediate enhancement behavior.

**Table 4:** Quantitative and semiquantitative imaging parameters by lesion type.

Parameter	Warthin Tumor	Pleomorphic Adenoma	Other Benign Lesions *	Malignant Lesions
ADC (×10^−3^ mm^2^/s)	0.88 (0.78–0.99)	1.59 (1.44–1.77)	1.17 (1.03–1.31)	0.95 (0.83–1.11)
TTP (s)	49.8 (42.1–58.9)	181.4 (159.2–204.6)	123 (95–151)	62.0 (52.4–78.6)
WR (%)	58 (51–65)	4 (1–8)	22 (12–32)	32 (23–42)
AUC (mM·s)	96 (83–109)	62 (49–75)	78 (60–92)	85 (71–100)
Kep (×10^−3^/min)	998 (870–1135)	354 (253–481)	509 (321–702)	480 (390–605)
Ve (×10^−3^)	121 (97–145)	280 (210–340)	261 (203–332)	245 (203–300)
Ktrans (×10^−3^/min)	212 (170–274)	140 (104–184)	172 (138–210)	188 (145–238)
Vp (×10^−3^)	75 (59–92)	50 (37–65)	58 (43–72)	62 (49–80)

Note. Continuous variables are reported as median (interquartile range, IQR), except for other benign lesions * (*n* = 3), which are reported as median (minimum–maximum) owing to the limited sample size of this subgroup. ADC indicates apparent diffusion coefficient; TTP, time-to-peak; WR, wash-out ratio; AUC, area under the enhancement curve; Ktrans, volume transfer constant; Kep, reflux rate constant; Ve, extravascular extracellular volume fraction; and Vp, plasma volume fraction. Statistically significant differences between Warthin tumors and malignant lesions were observed for Kep (*p* = 0.002) and Ve (*p* = 0.004), whereas Ktrans and Vp showed no statistically significant differences (all *p* > 0.05).

**Table 5:** Diagnostic performance of selected imaging parameters for lesion differentiation.

Parameter	Comparison	ROC AUC	Threshold	Sensitivity (%)	Specificity (%)
Kep	Warthin tumor vs. malignant lesions	0.96	723 × 10^−3^/min	92.3	93.3
Ve	Warthin tumor vs. malignant lesions	0.87	180 × 10^−3^	80.8	86.7

Note. Diagnostic performance metrics include area under the ROC curve, optimal threshold, sensitivity, and specificity. Warthin tumors were defined as the positive class (Kep ≥ threshold, Ve ≤ threshold). Corresponding 95% confidence intervals are reported in the Section 3.

## Data Availability

The data presented in this study are available on request from the corresponding author due to institutional, privacy, and ethical restrictions.

## Abbreviations

ADC	Apparent diffusion coefficient
AIF	Arterial input function
AUC	Area under the curve
DCE-MRI	Dynamic contrast-enhanced magnetic resonance imaging
DWI	Diffusion-weighted imaging
FA	Flip angle
ICC	Intraclass correlation coefficient
IQR	Interquartile range
IRB	Institutional Review Board
Kep	Reflux rate constant
Ktrans	Volume transfer constant
MRI	Magnetic resonance imaging
PACS	Picture Archiving and Communication System
ROC	Receiver operating characteristic
ROI	Region of interest
SI	Signal intensity
STIR	Short tau inversion recovery
TIC	Time–intensity curve
TTP	Time-to-peak
Ve	Extravascular extracellular volume fraction
Vp	Plasma volume fraction
WR	Wash-out ratio

## References

[1] Abdel Razek A.A.K., Mukherji S.K. (2018). State-of-the-Art Imaging of Salivary Gland Tumors. Neuroimaging Clin. N. Am..

[2] Maraghelli D., Pietragalla M., Cordopatri C., Nardi C., Peired A.J., Maggiore G., Colagrande S. (2021). Magnetic Resonance Imaging of Salivary Gland Tumours: Key Findings for Imaging Characterisation. Eur. J. Radiol..

[3] Christe A., Waldherr C., Hallett R., Zbaeren P., Thoeny H. (2011). MR Imaging of Parotid Tumors: Typical Lesion Characteristics Improve Discrimination between Benign and Malignant Disease. Am. J. Neuroradiol..

[4] Tartaglione T., Botto A., Sciandra M., Gaudino S., Danieli L., Parrilla C., Paludetti G., Colosimo C. (2015). Differential Diagnosis of Parotid Gland Tumours: Which MR Findings Should Be Taken into Account?. Acta Otorhinolaryngol. Ital..

[5] Kim S.Y., Borner U., Lee J.H., Wagner F., Vogel D.W.T. (2022). Magnetic Resonance Imaging of Parotid Gland Tumors: A Pictorial Essay. BMC Med. Imaging.

[6] Coudert H., Mirafzal S., Dissard A., Boyer L., Montoriol P.-F. (2021). Multiparametric Magnetic Resonance Imaging of Parotid Tumors: A Systematic Review. Diagn. Interv. Imaging.

[7] Gökçe E. (2020). Multiparametric Magnetic Resonance Imaging for the Diagnosis and Differential Diagnosis of Parotid Gland Tumors. J. Magn. Reson. Imaging.

[8] Eida S., Sumi M., Sakihama N., Takahashi H., Nakamura T. (2007). Apparent Diffusion Coefficient Mapping of Salivary Gland Tumors: Prediction of Benignancy and Malignancy. Am. J. Neuroradiol..

[9] Yerli H., Aydin E., Haberal N., Harman A., Kaskati T., Alibek S. (2010). Diagnosing Common Parotid Tumours with MRI Including Diffusion-Weighted Imaging versus Fine-Needle Aspiration Cytology. DentoMaxilloFac. Radiol..

[10] Mikaszewski B., Markiet K., Smugała A., Stodulski D., Szurowska E., Stankiewicz C. (2017). Diffusion- and Perfusion-Weighted MRI in Differentiation of Benign and Malignant Parotid Tumors. J. Oral Maxillofac. Surg..

[11] Zaghi S., Hendizadeh L., Hung T., Farahvar S., Abemayor E., Sepahdari A.R. (2014). MRI Criteria for the Diagnosis of Pleomorphic Adenoma: A Validation Study. Am. J. Otolaryngol..

[12] Connolly M., Srinivasan A. (2018). Diffusion-Weighted Imaging in Head and Neck Cancer: Technique, Limitations, and Applications. Magn. Reson. Imaging Clin. N. Am..

[13] Yabuuchi H., Matsuo Y., Kamitani T., Setoguchi T., Okafuji T., Soeda H., Sakai S., Hatakenaka M., Nakashima T., Oda Y. (2008). Parotid Gland Tumors: Can Addition of Diffusion-Weighted MR Imaging Improve Dynamic MR Imaging Diagnosis?. Radiology.

[14] Yuan Y., Tang W., Tao X. (2016). Parotid Gland Lesions: Separate and Combined Diagnostic Value of Conventional MRI, Diffusion-Weighted Imaging and Dynamic Contrast-Enhanced MRI. Br. J. Radiol..

[15] Zheng N., Li R., Liu W., Shao S., Jiang S. (2018). Diagnostic Value of Combining Conventional MRI, DWI and DCE-MRI for Salivary Gland Tumors. Br. J. Radiol..

[16] Xu Z., Zheng S., Pan A., Cheng X., Gao M. (2019). A Multiparametric Analysis Based on DCE-MRI to Improve the Accuracy of Parotid Tumor Discrimination. Eur. J. Nucl. Med. Mol. Imaging.

[17] Pietragalla M., Nardi C., Bonasera L., Mungai F., Taverna C., Novelli L., De Renzis A.G.D., Calistri L., Tomei M., Occhipinti M. (2020). The Role of Diffusion-Weighted and Dynamic Contrast Enhancement Perfusion-Weighted Imaging in the Evaluation of Salivary Glands Neoplasms. Radiol. Med..

[18] Yabuuchi H., Kamitani T., Sagiyama K., Yamasaki Y., Hida T., Matsuura Y., Hino T., Murayama Y., Yasumatsu R., Yamamoto H. (2020). Characterization of Parotid Gland Tumors: Added Value of Permeability MR Imaging to DWI and DCE-MRI. Eur. Radiol..

[19] Huang N., Chen Y., She D., Xing Z., Chen T., Cao D. (2022). Diffusion Kurtosis Imaging and DCE-MRI for Differentiation of Parotid Gland Tumors. Eur. Radiol..

[20] Gökçe E., Beyhan M. (2023). Diagnostic Efficacy of Diffusion-Weighted Imaging and Semiquantitative and Quantitative Dynamic Contrast-Enhanced Magnetic Resonance Imaging in Salivary Gland Tumors. World J. Radiol..

[21] Jia C.H., Wang S.Y., Li Q., Qiu J.M., Kuai X.P. (2021). Conventional, Diffusion, and Dynamic Contrast-Enhanced MRI Findings for Differentiating Metaplastic Warthin’s Tumor of the Parotid Gland. Sci. Prog..

[22] Mungai F., Verrone G.B., Bonasera L., Bicci E., Pietragalla M., Nardi C., Berti V., Mazzoni L.N., Miele V. (2021). Imaging Biomarkers in the Diagnosis of Salivary Gland Tumors: The Value of Lesion/Parenchyma Ratio of Perfusion-MR Pharmacokinetic Parameters. Radiol. Med..

[23] Elmokadem A.H., Abdel Khalek A.M., Abdel Wahab R.M., Tharwat N., Gaballa G.M., Elata M.A., Amer T. (2019). Diagnostic Accuracy of Multiparametric Magnetic Resonance Imaging for Differentiation between Parotid Neoplasms. Can. Assoc. Radiol. J..

[24] Ogawa T., Kojima I., Ishii R., Sakamoto M., Murata T., Suzuki T., Kato K., Nakanome A., Ohkoshi A., Ishida E. (2018). Clinical Utility of Dynamic-Enhanced MRI in Salivary Gland Tumors. Eur. Arch. Otorhinolaryngol..

[25] Lechner Goyault J., Riehm S., Neuville A., Gentine A., Veillon F. (2011). Interest of Diffusion-Weighted and Gadolinium-Enhanced Dynamic MR Sequences for the Diagnosis of Parotid Gland Tumors. J. Neuroradiol..

[26] Shukla-Dave A., Obuchowski N.A., Chenevert T.L., Jambawalikar S., Schwartz L.H., Malyarenko D., Huang W., Noworolski S.M., Young R.J., Shiroishi M.S. (2019). Quantitative Imaging Biomarkers Alliance (QIBA) Recommendations for Improved Precision of DWI and DCE-MRI Derived Biomarkers in Multicenter Oncology Trials. J. Magn. Reson. Imaging.

[27] Kim H. (2018). Variability in Quantitative DCE-MRI: Sources and Solutions. J. Nat. Sci..

[28] Sourbron S.P., Buckley D.L. (2013). Classic Models for Dynamic Contrast-Enhanced MRI. NMR Biomed..

[29] Tofts P.S., Kermode A.G. (1991). Measurement of the Blood-Brain Barrier Permeability and Leakage Space Using Dynamic MR Imaging. 1. Fundamental Concepts. Magn. Reson. Med..

[30] Hagiwara A., Fujita S., Ohno Y., Aoki S. (2020). Variability and Standardization of Quantitative Imaging: Monoparametric to Multiparametric Quantification, Radiomics, and Artificial Intelligence. Investig. Radiol..

